# Sarcopterygian fin ontogeny elucidates the origin of hands with digits

**DOI:** 10.1126/sciadv.abc3510

**Published:** 2020-08-19

**Authors:** Joost M. Woltering, Iker Irisarri, Rolf Ericsson, Jean M. P. Joss, Paolo Sordino, Axel Meyer

**Affiliations:** 1 Zoology and Evolutionary Biology, Department of Biology, Universität Konstanz, Universitätstrasse 10, 78464 Konstanz, Germany.; 2 Macquarie University, Sydney, NSW 2109, Australia.; 3 Department of Biology and Evolution of Marine Organisms, Stazione Zoologica Anton Dohrn, Villa Comunale, 80121 Naples, Italy.

## Abstract

How the hand and digits originated from fish fins during the Devonian fin-to-limb transition remains unsolved. Controversy in this conundrum stems from the scarcity of ontogenetic data from extant lobe-finned fishes. We report the patterning of an autopod-like domain by *hoxa13* during fin development of the Australian lungfish, the most closely related extant fish relative of tetrapods. Differences from tetrapod limbs include the absence of digit-specific expansion of *hoxd13* and *hand2* and distal limitation of *alx4* and *pax9*, which potentially evolved through an enhanced response to *shh* signaling in limbs. These developmental patterns indicate that the digit program originated in postaxial fin radials and later expanded anteriorly inside of a preexisting autopod-like domain during the evolution of limbs. Our findings provide a genetic framework for the transition of fins into limbs that supports the significance of classical models proposing a bending of the tetrapod metapterygial axis.

## Teaser

Lungfish fin development reveals the evolutionary origins of tetrapod limb patterning.

## INTRODUCTION

The functional units of hands and feet, located at the distal end of our limbs, are collectively composed of wrist/ankle bones, metacarpals/metatarsals (the middle hand/foot), and digits (fingers and toes). Together with the more proximal bones of the stylopod (upper arm/leg) and zeugopod (lower arm/leg), they represent a highly constrained *Bauplan* that originated at the base of the radiation of land vertebrates and defines the tetrapod lineage (*1*, *2*). Sarcopterygian fish, including the living lineages of the lungfishes and the coelacanth, as well as extinct tetrapodomorphs, show homologous structures in their fins to our proximal limb elements (*3*–*10*). However, the unambiguous identification of evolutionary precursors for more distal bones remains problematic. For this reason, functional hands and feet with digits have traditionally been considered to be an evolutionary key innovation—“the fin-to-limb transition”—that first arose in tetrapods during the conquest of land (*3*, *4*, *11*). Since the 19th century, various transformational theories have been proposed to explain the evolution of the distal limb (*3*, *4*, *11*, *12*), which in the last 25 years have become integrated with emerging insights from the fields of developmental biology and gene regulation (*10*, *13*–*18*). Today, competing mutually incompatible hypotheses propose that digits could result from adoption of a dermoskeletal genetic network by the distal endoskeleton following the evolutionary loss of fin rays (*19*) or alternatively, that they arose through the emergence of new forms of the *hox* gene regulation producing limb-specific gene expression domains (*13*, *14*). One reason for the controversy in this research program stems from its reliance on work conducted on ray-finned fish (actinopterygians, such as zebrafish or paddlefish) whose fins are very different from those of the sarcopterygian crown group (lobe-finned fish), the lineage from which tetrapods derived. A resolution of the alternative explanations on the origin of limbs therefore requires their testing using sarcopterygian fish species. This has been challenging since coelacanths inhabit inaccessible oceanic realms, while the African and South American lungfishes—the sister lineage to the tetrapods (*20*)—have strongly secondarily reduced fins. Here, we use the Australian lungfish (*Neoceratodus forsteri*) as the only tractable sarcopterygian fish model for this question (*8*) to analyze the expression domains of hand- and digit-related genes in developing fins.

## RESULTS

### *Hoxa13* expression defines an autopodial-like domain during lungfish fin development

**Fig. 1:**
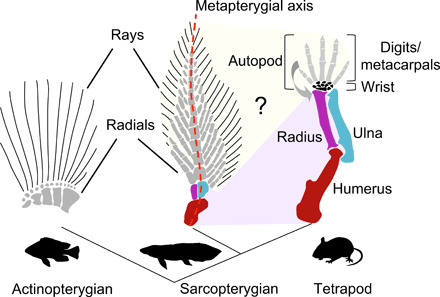
Homology between fins and tetrapods limbs. Sarcopterygian fins (Australian lungfish fin shown) resemble tetrapod limbs, and proximally clear homologs of the humerus, radius, and ulna can be identified (pink field) (*5*, *8*), an organization that is absent from ray finned fish (actinopterygians). In the distal region, they, however, lack the tetrapod-specific cross-articular anatomy (bend arrow) by which the long bones of the hand articulate with the radius and ulna via the nodular bones of the wrist (black). Hence, the evolutionary origin of the hand and digits at the fin-to-limb transition remains unresolved (yellow field).

**Fig. 2:**
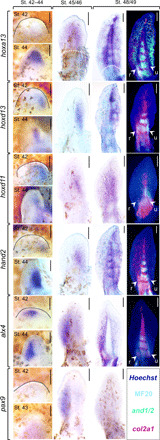
“Hand” and “digit” domains in embryonic pectoral fins of the Australian lungfish. Gene expression was detected using whole-mount in situ hybridization during stages 42 to 48/49. Names for colorimetrically detected genes are indicated on the left. Fluorescent detection is shown for the oldest stage in the rightmost column with detection for *col2a* in red, the anti-muscle sarcomere antibody MF20 in azure, *actinodin1/2* in green (performed for *hoxa13* sample only), and fin contours are shown using Hoechst staining (dark blue). The proximal boundary of *hoxa13* expression is indicated using a white dotted line. The radius (“r”) and ulna (“u”) are indicated in the rightmost column. Stages 45 to 49 fins were dissected and flat mounted, whereas stages 42 to 44 fins were imaged in position on the embryo. Additional expression data are provided in figs. S1 and S2. Anterior is to the left. Scale bars, 200 μm.

Australian lungfish fins have the typical sarcopterygian *Bauplan* characterized by a central metapterygial axis at the base of which homologs of the humerus, radius, and ulna can be distinguished (Fig. 1) (*5*, *8*). More distally, in the region of distal radials and rays, the homology with the limb remains controversially discussed (Fig. 1). In tetrapods, formation of the hand domain is governed by *hoxa13*, and its expression marks the boundary between the zeugopod (radius/tibia and ulna/fibula) and the autopod (hand/foot) (*1*, *21*). In ray-finned fish, expression occurs primarily in the developing dermal skeleton (*19*, *22*, *23*), tentatively suggesting that digits might derive from fin rays. However, the possibility that an endoskeletal autopodial domain was already in place in the tetrapod ancestor remains untested due to a lack of data from sarcopterygian species. Using whole-mount in situ hybridization, we detect first expression of *hoxa13* posteriodistally in lungfish pectoral fins at stages 42 to 44 (Fig. 2 and fig. S1), which at stage 45 has developed into a domain covering most of the fin except for the fin base (Fig. 2). At stage 47, a strong expression is observed in the condensing elements of the metapterygial axis (fig. S2), and at stage 48/49 also appears in the lateral domains where distal radials and rays will form (Fig. 2). To unambiguously distinguish between the endochondral and dermal fin domains, we used multicolor labeling for *actinodin1/2* to visualize the fin fold [which foreshadows the ray domain (*17*, *22*)] in combination with *collagen2a1* and an anti-sarcomere antibody to label the endochondral skeleton with its musculature. This further confirms that the *hoxa13* expression delimits the endochondral part of the skeleton distal from the radius and ulna (Fig. 2 and fig. S2); that is, at the same position along the proximodistal axis as in developing tetrapod limbs and its expression thus demarcates an autopod-like domain in the distal fin endoskeleton.

### Different posteriorization of lungfish fins

To evaluate these findings within the context of limb evolution, we note that tetrapod hands (as well as feet) are compound structures composed of the nodular bones of the mesopodium and the long bones of the digits and metacarpals. Together, these provide the distal unit of the highly constrained tetrapod “cross-articular” *Bauplan* that allows the hand palm to articulate with the radius and ulna via the wrist (Fig. 1) (*1*, *2*, *9*). Whereas, the expression of *hoxa13* suggests that the “hand” domain has a pre-tetrapod origin, such cross-articulating arrangement of long bones interspersed by nodular bones is not found in any fin skeleton, including that of sarcopterygians. This morphological dissonance thus implies that the fish distal fin domain underwent substantial changes along its proximal-distal axis at the evolutionary transition from fins to limbs during the evolution of the tetrapod autopod. In this regard, it is relevant that the patterning processes for the proximal-distal and anterior-posterior axes of the tetrapod hand are deeply interwoven (*24*) and that key genes driving the development of the distal limb have a posteriorly originating expression domain. *Hoxa13*, *hoxd13*, and *hand2* initiate their expression posteriorly in the early limb bud, and it is only during subsequent outgrowth that their domains become anteriorly expanded to occupy the distal limb margin (*21*, *25*–*27*). At the same time, anterior markers, such as *alx4* and *pax9*, do not extend distally and become excluded from the hand (*28*–*30*). Therefore, a phase of “progressive posteriorization” confers a posteriorly originating identity onto the most distal aspect of our limbs. To better understand their proximal-distal patterning, we analyzed the occurrence of this process in lungfish fins. *Hoxd13* and *hand2* become activated in a posterior domain at stage 42/43 (Fig. 2 and fig. S1) similar to their expression in mouse limb buds. At stages 44 to 46, during elongation of the fin bud, these domains become distally extended within the posterior fin (Fig. 2 and fig. S1), and at stages 48 to 50, they extend throughout the posterior fin in parallel with the metapterygial axis (Fig. 2 and fig. S2). During this process, *hand2* expression becomes slightly expanded anteriorly, whereas *hoxd13* maintains its approximate anterior-posterior expression boundary. Both genes occupy a posterior territory parallel to the metapterygial axis, resulting in an anterior domain in which they are not expressed [note that we do not detect an anterior to posterior progression of *hoxd13* expression, as was suggested previously (*8*); also see Supplementary Materials and Methods]. On this opposite side of the metapterygial axis, the anterior marker *alx4* (*31*) shows a spatiotemporal progression similar to the posterior genes, thereby creating a near mirror-image domain (Fig. 2). Expression of the anterior gene *pax9* (*30*) is detected only around stage 45 and is expressed similarly to *alx4* in an anterior territory that extends along the proximal-distal aspect of the metapterygial axis (Fig. 2).

In tetrapod limbs, a further manifestation of its posteriorization is the reverse-collinear expression of *hoxd* genes in the long bones of the hand. In the early limb bud, these genes are activated collinearly, whereby *hoxd11* is expressed more anteriorly than *hoxd13*, a relationship that subsequently becomes reversed in the distal domain during a second phase of regulation (*1*, *21*, *26*). Analysis of *hoxd11* expression (Fig. 2) shows that, at stages 42 to 48, *hoxd13* is not expressed more anteriorly than *hoxd11*, indicating the absence of reverse-collinear *hoxd* expression during lungfish fin development.

### Characterization of the lungfish ZPA

**Fig. 3:**
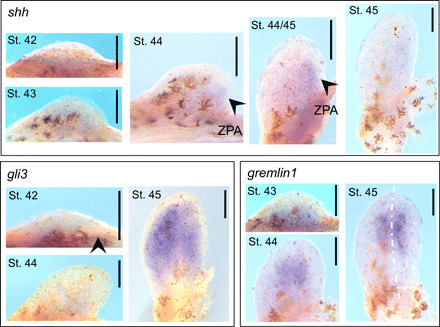
Expression of *shh*, *gli3*, and *gremlin1* during lungfish development. *Shh* expression is observed in a posterior ZPA during stage 44 (black arrowhead) but subsides at stage 45. *Gli3* is detected in a weak domain with anterior bias during fin budding (stage 42), leaving a clear posterior domain (black arrowhead) in which it is not expressed, and its expression is hence complementary with the early expression of *hand2* (Fig. 2) similar to what is observed in tetrapod limbs (*32*). Expression of the BMP antagonist *gremlin1* becomes expressed in a central fin domain along both anterior and posterior aspects of the forming metapterygial axis (dotted line in stage 45). Fins were dissected and imaged on an agarose dish. Additional data are provided for stages 42 to 45 in fig. S1. Anterior is to the left. Scale bars, 200 μm.

In tetrapod limbs, the phase of progressive posteriorization is established through the activity of *sonic hedgehog* (*shh*) secreted from the zone of polarizing activity (ZPA), which antagonizes the repressive action of the transcription factor *gli3* (*24*, *32*–*34*). This *shh* signal is required for the anterior expansion of *hoxa13*, *hoxd13*, *hoxd11*, and *hand2* (*33*–*35*) and for the concomitant reduction of the anterior *alx4* and *pax9* domains (*30*, *31*). In the fins of the Australian lungfish, *shh* expression is first detected at very low levels throughout the posterior fin around stage 43 and develops into a posterior ZPA that is present from stages 44 and 45 (Fig. 3), after which *shh* expression becomes undetectable. Expression of *gli3*, which, in turn, determines anterior limb identity (*30*, *31*, *33*–*36*), is detected at low intensity from stages 42 to 44 and increases strongly throughout the fin afterward at stage 45 (Fig. 3). At stage 42, *gli3* is expressed with the same anterior bias as in the early mouse limb (*32*).

The maintenance of the ZPA during limb development depends on a feedback loop whereby *shh* activates the secreted BMP inhibitor *gremlin1*, which is required for *fgf* expression in the apical ectodermal ridge (AER) (*37*, *38*). Sustained fibroblast growth factor (FGF) signaling by the posterior AER, in turn, provides a signal for ZPA survival. *Gremlin1* expression in Australian lungfish fins was detected in a central domain similar to that observed in tetrapod limbs, present on both anterior and posterior sides of the metapterygial axis originating posteriorly, consistent with its activation by *shh* (Fig. 3 and fig. S1). *Fgf8* expression in the posterior AER of Australian lungfish fins has been reported from early fin budding until the transition of the AER into an apical ectodermal fold (AEF) around stage 45 (*39*), coinciding with the disappearance of the ZPA and *shh* expression around this stage. Therefore, the components of the *shh-gli3* axis and the *shh-gremlin1-fgf8* feedback loop are expressed in a manner consistent with a role in anterior-posterior patterning similar to that in tetrapod limbs, although perhaps with different dynamics that could explain the different posteriorization of lungfish fins (see below).

## DISCUSSION

### An incomplete posteriorization of the distal domain in fins

**Fig. 4:**
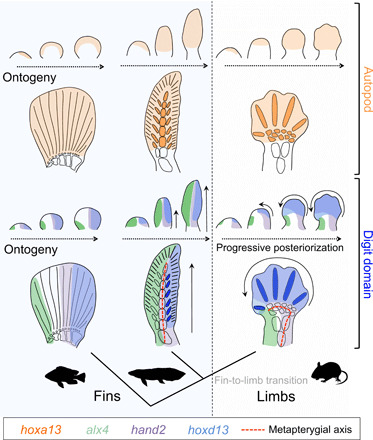
A compound origin for hands with digits. During development, *hoxa13* demarcates a similar endochondral autopodial domain in lungfish and tetrapods (top, orange), whereas this gene is primarily expressed in the dermal skeleton in ray-finned fish. Lungfish, however, lack the progressive posteriorization that patterns tetrapod digits (bottom, blue). Instead, genes such as *hand2* and *hoxd13* remain restricted to the posterior side of the fin, while an anterior domain patterned by *alx4*, which in tetrapods becomes excluded from the distal limb, extends along the proximal-distal fin axis. This terminal phase of posteriorization is therefore a distinguishing feature of tetrapods. Together, this suggests separated evolutionary trajectories for hand and digit domains and supports an inferred bending of the tetrapod metapterygial axis and homology between digits and postaxial fin radials (*3*, *4*, *48*). A generalized expression signature for ray-finned fish was reconstructed using (*12*, *22*, *23*) for *hoxa13*, (*12*, *42*) for *hoxd13*, (*18*, *43*) and fig. S3 for *hand2*, and (*43*) and fig. S3 for *alx4*. A teleost fin skeleton is shown as a generalized representation for ray-finned fish (also see main text).

Lungfish fins show strong resemblance to tetrapod limbs with respect to the proximal-distal patterning of their endoskeleton into an arm and hand domain by the posterior *hoxa* genes, whereby *hoxa11* marks the stylopod-zeugopod transition (*5*) and *hoxa13* marks the domain distal to the zeugopod. Therefore, an autopod-like domain that pre-dates the evolution of tetrapods is present in lungfish fins. At the same time, departures from limb-like proximal-distal gene expression exist, and these result from a different progression of the patterning of the anterior-posterior axis, whereby the lack of a complete phase of posteriorization translates into disparate gene expression profiles along the proximal-distal axis of limbs versus fins (Fig. 4). A diversity of morphological fin “archetypes” exists in fish. These exhibit, for instance, a reduction in the number of basal fin radials or, in teleosts, the loss of the metapterygium (*10*, *12*), whereby each of such transitions is likely related to alterations in the patterning of the anterior-posterior fin axis (*10*, *40*, *41*). Despite these differences, a generalized expression signature can be identified that consistently distinguishes fins from limbs. In fins, *hoxa13* occupies a distal domain along the entire anterior-posterior axis, but *hoxd13* and *hand2* remain confined to a more posterior domain and *alx4* and *pax9* are not distally limited (Fig. 4 and fig. S3) (*10*, *12*, *22*, *23*, *40*–*43*). The extension of a phase of progressive posteriorization in tetrapods therefore likely represents a derived heterochronic developmental mechanism that distinguishes limbs from fins.

The distal aspect of the limbs becomes posteriorized under the influence of *shh* secreted from the ZPA, and *shh* activates the same posterior genetic program in fish (*12*, *18*, *40*). However, the response in fins appears to be different from that in tetrapod limbs, as the expression of *hoxa13* and *gremlin1* becomes anteriorly displaced, whereas that of *hoxd13* and *hand2* remains posterior, and the expression of anterior markers *alx4* and *pax9* persists distally. Different temporal requirements for *shh* signaling have been demonstrated in limbs (*34*), whereby *gremlin1* is fully activated by brief stimulation, whereas the anteriorization of *hoxd13* depends on prolonged exposure. To our knowledge, *hoxa13*, *hand2*, *alx4*, and *pax9* have not been analyzed in this context yet, but on the basis of the available data, the patterns observed in fins are best interpreted as a developmentally truncated response to *shh*, resulting in an incomplete posteriorization of the distal domain as compared to limbs. The time window during which a clear ZPA is observed in lungfish fins is relatively short, and alternative mechanisms could be responsible for its termination in fins and limbs. In limbs, persistence of the ZPA depends on sustained FGF signaling from the AER, which is dependent on the bone morphogenetic protein (BMP) inhibition by *gremlin1* (*37*, *38*). In the Australian lungfish, both *fgf8* expression (*39*) and the ZPA disappear around the same time when the AER transforms into AEF upon the formation of the fin fold, and there is evidence that in fins, these events are related. In zebrafish, for example, the experimental perturbation of fin fold formation results in persisting *fgf8* expression (*16*) and an extended ZPA (*17*) as well as anteriorized *hoxd13* expression (*17*). This therefore suggests that the formation of the fin fold may be instrumental in the termination of the ZPA in fish fins, for instance, by acting as a physical barrier between epithelial FGF signaling and the mesenchymal ZPA. The evolutionary loss of the fin fold in tetrapods thus might contribute to a longer-lived ZPA during limb outgrowth, resulting in an increased posteriorization of distal limbs as compared to fish fins. Alternatively, the evolution of additional *hoxd* cluster regulation in tetrapods (*1*, *14*) could have contributed to an enhanced response to the posteriorizing *shh* signal. *Hoxd* genes in digits are regulated by a complex regulatory region containing numerous partially redundant enhancers (*44*). In addition to a quantitative decrease in expression levels, a posteriorly restricted *hoxd* expression pattern is observed in several deletion mutants affecting this region (*44*). A combination of enhancer dosage effects via the acquisition of novel *hoxd* enhancers, in combination with sustained *shh* signaling by the ZPA, might therefore cause the anteriorized *hoxd* gene expression in the distal tetrapod limb. Such a scenario concurs with the proposed role of quantitative and heterochronic shifts in the *shh/gli3* axis as a driver of the morphological divergence in paired appendages (*10*, *40*, *41*).

### A transformational scenario for the fin-to-limb transition

Although an autopod-like domain forms during the proximal-distal differentiation of lungfish fins, this domain is morphologically different from that in tetrapod limbs and fails to complete the progressive posteriorization, as is typically associated with the formation of digits. Whereas the function of *hoxa13* is linked to the entire hand domain and might specifically contribute to the nodular character of the mesopodium, the distal expression of *hoxd13* is intrinsically related to the long-bone territory of the hand comprising the metacarpals/metatarsals and digits (*1*). The different expression dynamics of these genes in lungfish fins therefore indicate separated evolutionary and developmental trajectories for hand and digit domains, as is also suggested by paleontological data on the morphological sequence of distal fin evolution in sarcopterygians (*11*). In this scheme, an ancestral autopod-like field defined by distal *hoxa13* pre-dates the evolution of digits and was coopted in tetrapods to form the hands and feet. The concomitant emergence of digits as neomorphic structures (*1*, *2*, *13*, *45*) likely required additional changes in the genetic modules downstream of the posteriodistal genes analyzed here in the *Neoceratodus* fins. Further comparative analysis of digit-specific gene regulatory networks (*46*, *47*) in fins will therefore yield insights into the molecular and developmental mechanisms that shaped the fin-to-limb transition.

Our findings do have important implications for how we interpret the morphological changes that occurred at the fin-to-limb transition and how the hand and digit domains originated during evolution. In the last century, several competing transformational hypotheses have been proposed for the transition of fins into limbs. One influential model, eloquently articulated by Shubin and Alberg (*48*) but also presented by earlier authors in simpler form (*3*, *4*, *45*), predicts that digits arose from postaxial fin radials through their translocation to the distal domain by a bending of the metapterygial axis. This model has its limitations (*49*), and no such actual bending or causative cell movements have been demonstrated during tetrapod limb development. These morphological changes therefore likely reflect the transcriptional respecification of the anterior domain, thereby creating the illusion of metapterygial “bending.” We report differences in the patterning of fins and limbs that show a remarkable congruence with this inferred anterior respecification. In the lungfish, *hoxd13* is restricted to the domain from which postaxial fin radials will develop, whereas *hoxa13* is expressed more widely in the central elements of the metapterygial axis. Consequently, the presence and absence of a phase of distal expansion of *hoxd13* correlate with bent versus straight metapterygial axes in Australian lungfish and tetrapods, respectively. Our results thus indicate that the most parsimonious identification of “digit precursors” in sarcopterygian fish lies in postaxial fin radials (Fig. 4).

Australian lungfish have the same basic anterior-posterior skeletal organization as the extinct lineage of tetrapodomorphs represented by *Tiktaalik* (*7*), whereby distal radials are present on preaxial and postaxial sides of a central axis. The distal fin radials of *Elpistostege*, a recently described more crownward tetrapodomorph, have been identified as digit precursors and are more tetrapod-like in their presence on the distal fin margin (*50*). Although, these still have a postaxial bias and do not cross-articulate with the radius as in tetrapods. This thus suggests that the posteriorization of the distal paired appendages evolved close to, or coinciding with, the origin of tetrapods and was involved in a widening and anterior expansion of the distal radial or digit domain. We propose that the derived *Bauplan* of tetrapod limbs, i.e., with the digits juxtaposed to the lower arm in a cross-articulating position, arose from an ancestral autopod-like domain that became modified through the extension of a phase of developmental posteriorization. In this form, limbs with coherent hands and feet provided our ancestors 400 million years ago with an adaptation to conquer the land and, remarkably, have stayed with us ever since as the most distinguishing feature of the tetrapod radiation.

## MATERIALS AND METHODS

### In situ hybridization

In situ hybridization was performed according to Woltering *et al*. (*51*), with modifications (see Supplementary Materials and Methods).

### Probe cloning

Probes were amplified from cDNA or genomicDNA using Taq polymerase and subsequently cloned in the pGEMT vector (Promega A3600). Gene-specific primers are given in table S1. For all lungfish probes shown in Figs. 2 and 3 and fig. S2 (except *hand2*, *shh*, *pax9*, and *gremlin1*), two probes spanning different regions of the gene were cloned to increase the total span used in the in situ hybridization experiments. A separate probe set was used for the in situ hybridization experiments shown in fig. S1 (see Supplementary Materials and Methods).

### Experimental animal protocols

Australian lungfish staging was according to Kemp (*52*). Larvae were obtained, as described before (*39*), and were collected between stages 42 (3 to 4 weeks after fertilization) and 50 (10 to 12 weeks after fertilization). All lungfish care and experimental procedures were approved by the Animal Research 347 Authority (ARA) at Macquarie University (ARA 2009/039). *Astatotilapia burtoni* embryos were obtained before, as described in (*53*) under permit *Az.* #T15/05TFA.

### Image acquisition

Imaging was carried out using a Leica MZ10F binocular stereomicroscope equipped with either a DMC2900 camera (color) or a DFC3000G camera (fluorescent) and LAS v4.5 software using the Z-stacking option. Fluorescent images were constructed as overlays using false colors. Images were enhanced using brightness, contrast, and sharpening settings in Adobe Photoshop software. Figures 2 and 3 and fig. S2 show left or right fins imaged with the ventral side up oriented to place their anterior/preaxial side to the left (i.e., right fins were mirrored). All probes were analyzed on a minimum of two specimens per stage shown, and no significant differences were observed between left and right fins.

### Identification of gene orthologs in the Australian lungfish

A lungfish embryonic RNA sequencing transcriptome was assembled from the RNA extracted from embryonic fin tissue (see Supplementary Materials and Methods). Target transcripts were identified using Basic Local Alignment Search Tool (BLAST) with coelacanth, spotted gar, or *Xenopus* orthologous sequences as query. Phylogenetic analysis (see Supplementary Materials and Methods) was performed using orthologs and paralogs from human, spotted gar, elephant shark, and Australian lungfish to confirm correct orthology assignment (gene trees are shown in figs. S4 to S11).

## Supplementary Material

abc3510_SM.pdf

## SUPPLEMENTARY MATERIALS

Supplementary material for this article is available at http://advances.sciencemag.org/cgi/content/full/6/34/eabc3510/DC1

## Appendix

View/request a protocol for this paper from *Bio-protocol*.
