# Supplementary material for: Sarcopterygian fin ontogeny elucidates the origin of hands with digits
Source: Sci Adv. 2020 Aug 19;6(34):eabc3510. doi: 10.1126/sciadv.abc3510 (PMC7438105; doi:10.1126/sciadv.abc3510)
Supplement: abc3510_SM.pdf [file abc3510_SM.pdf]

Science Advances
AAAS

advances.sciencemag.org/cgi/content/full/6/34/eabc3510/DC1

Supplementary Materials for

Sarcopterygian fin ontogeny elucidates the origin of hands with digits

Joost M. Woltering*, Iker Irisarri, Rolf Ericsson, Jean M. P. Joss, Paolo Sordino, Axel Meyer

*Corresponding author. Email: joost.woltering@uni-konstanz.de, jmwoltering@gmail.com

Published 19 August 2020, *Sci. Adv.* **6**, eabc3510 (2020)
DOI: 10.1126/sciadv.abc3510

This PDF file includes:

Supplementary Materials and Methods
Figs. S1 to S11
Table S1
References

Supplementary Materials and Methods

In situ hybridization

In situ hybridization was performed according to Woltering et al. 2009 (*51*) and Lauter et al. 2011 (*54*) with the following modifications (except in situ experiments shown in **Fig.S1**, see below). Probe penetration has been reported as a major problem for performing *in situ* hybridization on lungfish larvae (*8*). Previous studies partially overcame this problem by substituting permeabilization using 50%MetOH/50% DMSO for the standard ProtK treatment, but even under such conditions were not able to use probe spans longer than 179bp (*8*). Use of short probe spans in general is expected to reduce the signal to noise ratio in the experiment (when adhering to a fixed absolute amount of RNA probe added to the hybridization step) because the final signal will be proportional to the amount of hapten (i.e. DIG, fluorescein or DNP) bound to the targeted transcript. Therefore, longer probe spans are expected to result in an increased signal. A potential trade-off here is that penetration of large RNA molecules into the cells can be an issue, particularly in tissue where permeabilization is already problematic as has bee reported for lungfish larvae. To deal with this problem, we further optimized the protocol by two more steps in addition to the overnight permeabilization step using 50%MetOH/DMSO as described (*8*). Firstly, we maintained the standard ProtK treatment using 10µg/ml in TBST for 25 minutes at RT for all stages investigated. Secondly (and most importantly), to optimize probe penetrance and allow the use of larger probe spans, we used controlled probe hydrolysis as described (*55*) by incubating of the total probe yield in 50ul 40mM $NaHCO_3$/60mM $NaCO_3$ for 20 minutes at 60°C. This step degrades longer probes into ~300nt fragments and allows penetration of larger probe spans as shorter fragments. This strategy of using longer but fragmented probes maintains the higher coverage of the targeted transcript (resulting in more hybridized hapten), while at the same time allowing probe penetration due to the reduced size. Using this strategy we were able to detect *hoxd13* expression at stages for which previously used protocols failed (*8*). The difference in *in situ* hybridization strategy likely explains the differing results obtained by us.

Combined colorimetric and fluorescent in situ hybridization was performed as follows: probes for all colorimetrically detected genes were synthesized using DIG-RNA-labeling-mix (Roche #11277073910), detected with α-DIG-AP (Roche # 11093274910), diluted 1:4000), and developed using BMpurple (Roche # 11442074001); the probes for *actinodin1/2* (*and1/2*) were synthesized using fluorescein-RNA-labeling-mix (Roche # 11685619910), detected with α-fluorescein-POD (Roche # 11426346910), diluted 1:500), and developed using bench made FITC-tyramide (*54*); the probes for *collagen2a1* (*col2a1*) were synthesized using DNP RNA labeling mix containing 3.5mM DNP-11-UPT (PerkinElmer, NEL555001EA,), 6.5nM UTP and 10mM of ATP, CTP and GTP each (TFS, R0481), detected using α-DNP-HRP (PerkinElmer FP1129, 1:100 in PBST, 10% FCS), and developed using bench made TAMRA-tyramide (*54*).
In the triple in *situs* for *hoxa13*, *and1/2* and *col2a1* the immuno step was performed simultaneously for α-DIG-AP and α-fluo-POD and the POD was developed first using FITC-tyramide followed by washes in AP buffer and detection of the αDIG-AP antibody using BMpurple. Subsequently the POD was inactivated using 10mM glycine pH2.0 followed by immuno using αDNP-POD, which was developed using TAMRA-tyramide. Double *in situs* for *alx4*, *hand2*, *hoxd13*, *hoxd11* combined with *col2a1* were first incubated in immuno for anti

αDIG-AP and developed using BMpurple, followed by a second immuno step using αDNP-HRP, which was developed using TAMRA-tyramide. Muscle sarcomeres were detected using α-myosin MF20 antibody (*56*) obtained from the DSHB, Iowa, which was added 1/200 to the αDNP-POD incubation step. After development of the α-DNP-POD samples were incubated overnight with a secondary goat-α-mouse dylight-650 1:200 (TFS #SA5-10174) in PBST-10%FCS to visualize the muscle sarcomeres.

The *in situ* experiments shown in **Fig. S1** (done by RE) were performed according to Woltering et al. 2009 (*51*) with the following modifications. The ProtK treatment was performed using 30 μg/ml for 15 to 30 minutes depending on the stage: early stages requiring less time than later stages. The acetic anhydride/triethanolamine treatment step was omitted. An additional prolonged bleaching step using 1.5% H_2O_2 was included after staining was completed to remove all embryonic pigmentation.

Transcriptome sequencing and assembly

We used a single embryo of *Neoceratodus forsteri* at developmental stage 51/52 (*52*) stored in RNAlater (Ambion, Austin, TX, USA). Both pectoral fins and caudal fin were dissected. Total RNA was extracted with Trizol (Invitrogen, Carlsbad, CA, USA) according to the manufacturer's recommendations, treated with DNAse I and purified in spin columns (Qiagen, Hilden, Germany). Ribosomal RNA was depleted with human-mouse-rat RiboZero Kit (Illumina, San Diego, CA, USA) using 28 μl of starting RNA and purified with RNAeasy MinElute Kit (Qiagen) to a final volume of 10.5 μl. Quantification and integrity was assessed using a Ribogreen assay and Bioanalyzer 2100 (Agilent Technologies, Waldbronn, Germany). Individual libraries were prepared with the Illumina TruSeq RNA Stranded mRNA Sample Preparation Kit (Part# 15031047 Rev. E Oct 2013) following manufacturer's instructions. Protocol was started by incubating 4 μl of RiboZero-depleted mRNA with 13 μl FPF mix at 94ºC for 8 min prior to first strand cDNA synthesis. 13 PCR cycles were used. All barcoded libraries were pooled into one Illumina lane and sequenced using HiSeq2500 2x150 bp technology.
Raw reads were quality-trimmed with Trimmomatic v. 0.33 (*57*) with default settings and remaining polyA tails removed with prinseq (*58*) whenever homopolymers were >7. Success of ribosomal RNA depletion step was confirmed by low mapping rate of individual reads (6.14%) to nuclear and mitochondrial ribosomal RNA sequences of *N. forsteri*. Transcriptome was assembled de novo using Trinity (*59*) with default settings. Raw reads are available from the NCBI Short Read Archive under BioProject PRJNA417954.

Othology determination

The orthology of N. forsteri sequences were corroborated through phylogenetic analysis with homologs and paralogs from elephant shark (*Callorhinchus millii*), spotted gar (*Lepisosteus oculatus*) and human (*Homo sapiens*). Sequences were downloaded from ENSEMBL release 99, masked with PREQUAL (*60*) and aligned with mafft v7.427 (*61*). Phylogenetic inference was performed with maximum likelihood in IQTREE v. 1.6.10 ((*62*)) under BIC-selected substitution models and branch support estimated with ultra-fast bootstrapping and SH-like aLRT, each with 1000 pseudoreplicates.

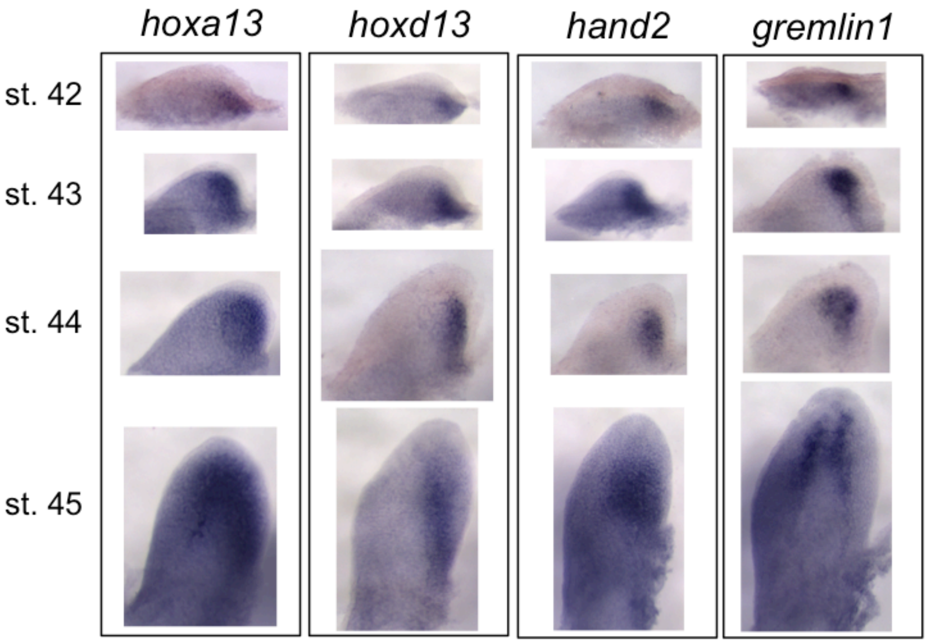

Fig. S1. Early expression of posterior genes during Australian lungfish fin development. Expression of *hoxa13*, *hoxd13*, *hand2* and *gremlin1* during early lungfish development. *Hoxd13* expression in the posterior fin but might be activated before *hoxa13*, as the latter is not always detected in the posterior fins of stage 42 embryos (N = 3 positive, 2 negative) (also see main **Fig. 2**). Fins were dissected and mounted for imaging. *Hoxa13* and *hoxd13* show a markedly different progression during early fin development coinciding with the expression of *shh* (main **Fig. 3**). Whereas the expression of both genes initiates posteriorly in the fin bud, only the expression of *hoxa13* becomes subsequently anteriorly expanded while *hoxd13* expression remains confined to a domain posterior of the metapterygial axis. *Hand2* shows some anterior expansion during early fin development but a domain anterior of the metapterygial axis persists where this gene is not expressed (also see main **Fig. 2**). *Gremlin1* expression starts posterior and expands anteriorly, consistent with activation by *shh* as known from limbs (*34*), and is at stage 45 present in a domain flanking the metapterygial axis on pre-axial as well as the post-axial sides (also see main **Fig. 3**). Fins were dissected and flatmounted before imaging. Anterior is to the left.

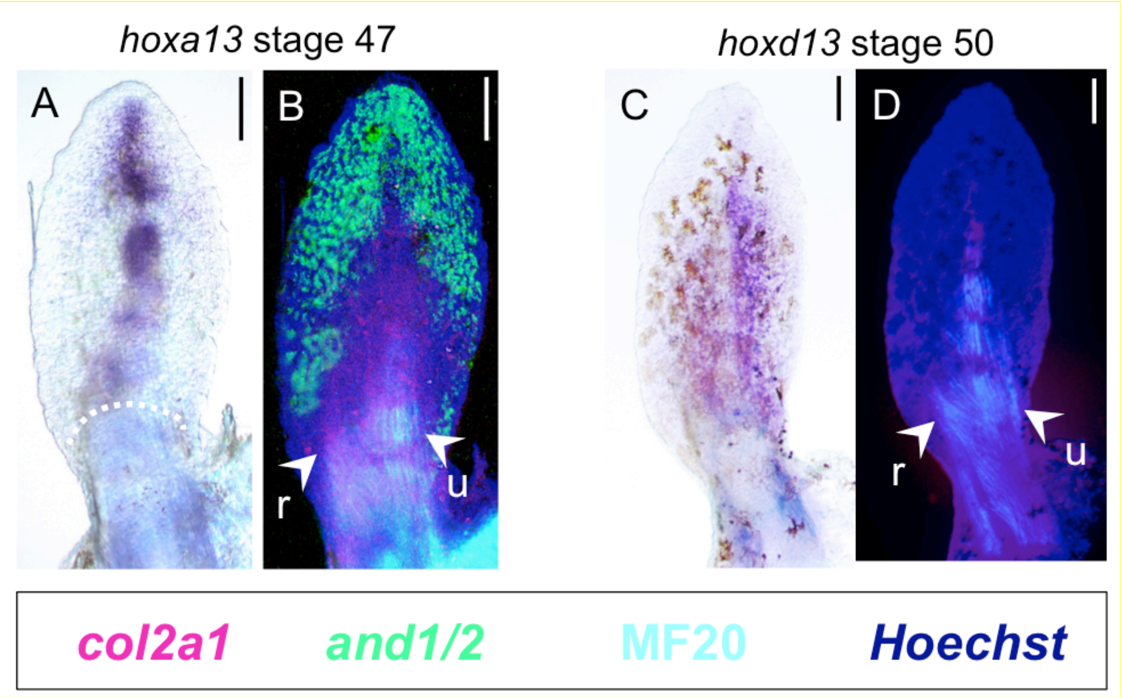

Fig. S2. Visualisation of "hand" and "digit" domains in developing pectoral fins of the Australian lungfish. Panel A and B, detection of *hoxa13* expression in a stage 47 fin. Colorimetric detection of *hoxa13* in panel A, the while dotted line indicates the proximal boundary of expression. Panel B shows fluorescent detection of *col2a1* (red), *actinodin1/2* (*and1/2*) (green) to label the fin fold and muscle sarcomere using MF20 antibody (azure). The radius ("r") and ulna ("u") are indicated in the panels showing fluorescent images. Staining for *hoxa13* can be observed in the condensing metapterygial elements distal of the radius and ulna. Panel C and D, detection of *hoxd13* in a stage 50 fin. Colorimetric detection of *hoxd13* in panel C. Panel D shows fluorescent detection of *col2a1* (red), muscle sarcomere using MF20 antibody (azure). Note that *hoxd13* expression shows a strong bias towards the post-axial fin mesenchyme. Hoechst staining was used to visualize the fin outlines in panel B and D. Fins were dissected and flatmounted before imaging. Anterior is to the left. Scale bars are 200 µm.

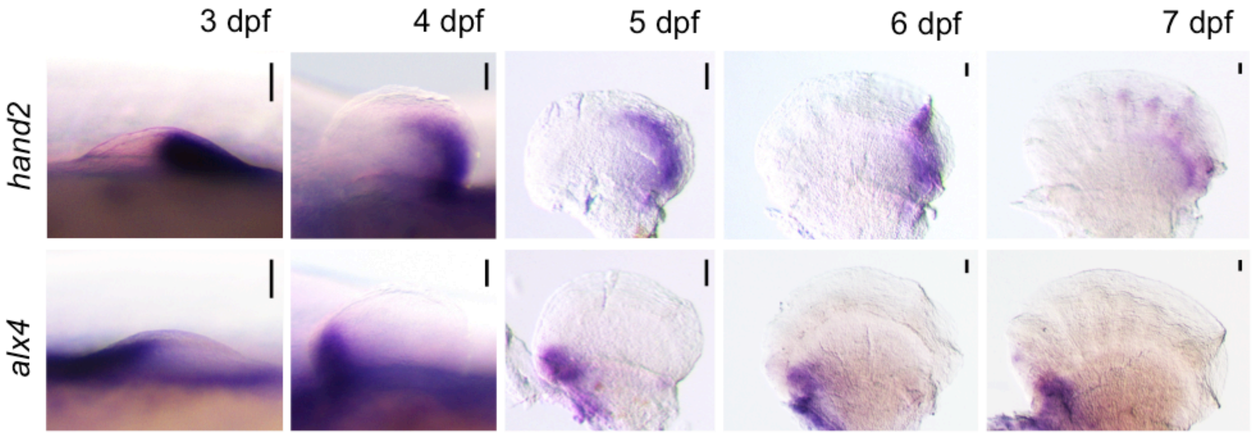

Fig. S3. Visualisation of *hand2* and *alx4* expression during *Astatotilapia burtoni* pectoral fin development. The developmental progression of the posterior fin marker *hand2* occurs in a proximal-distal manner and does not show the strong anterior expansion characteristic for tetrapods. The expression of the anterior fin marker *alx4* becomes extended throughout the proximo-distal extent of the fin, including the fin ray condensations. Data for adult zebrafish describing expression of *alx4* and *hand2* also suggest a similar progression throughout ontogeny (*43*). Stage 3 and 4 dpf fins were imaged in position on the embryo, while 5-7 dpf fins were dissected and flatmounted before imaging. Anterior is to the left. Scale bars are 50 µm**.**

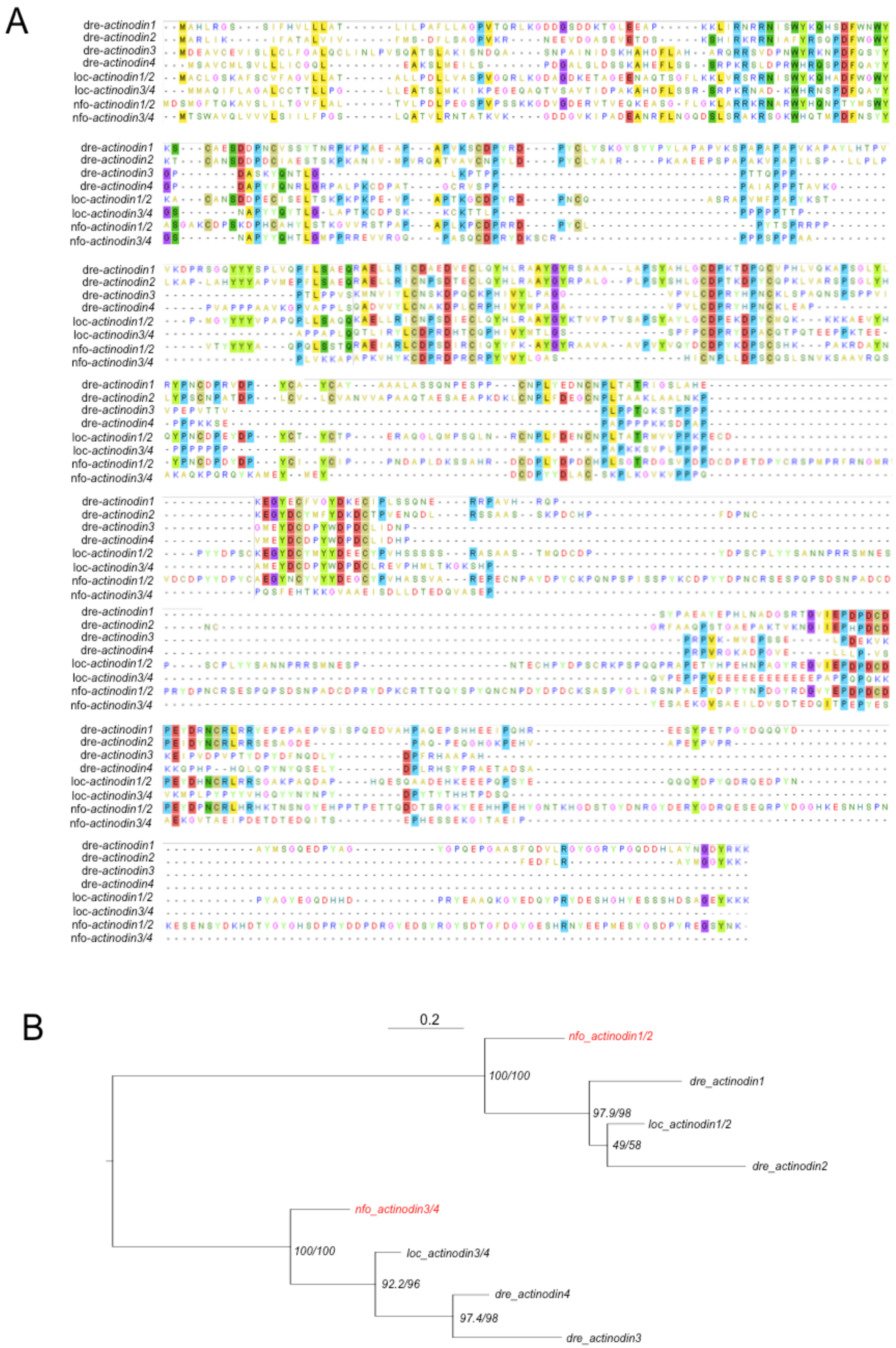

Fig. S4. Identification of *actinodin* gene sequences in the Australian lungfish. A) Alignment of zebrafish (dre), spotted gar (loc) and Australian lungfish (nfo) sequences. B) Gene tree confirming correct orthology assignment of the Australian lungfish *actinodin1/2* and *actinodin3/4* gene identities. Branch lengths are expected replacements per site and numbers at nodes ultrafast bootstrap and SH-like aLRT support.

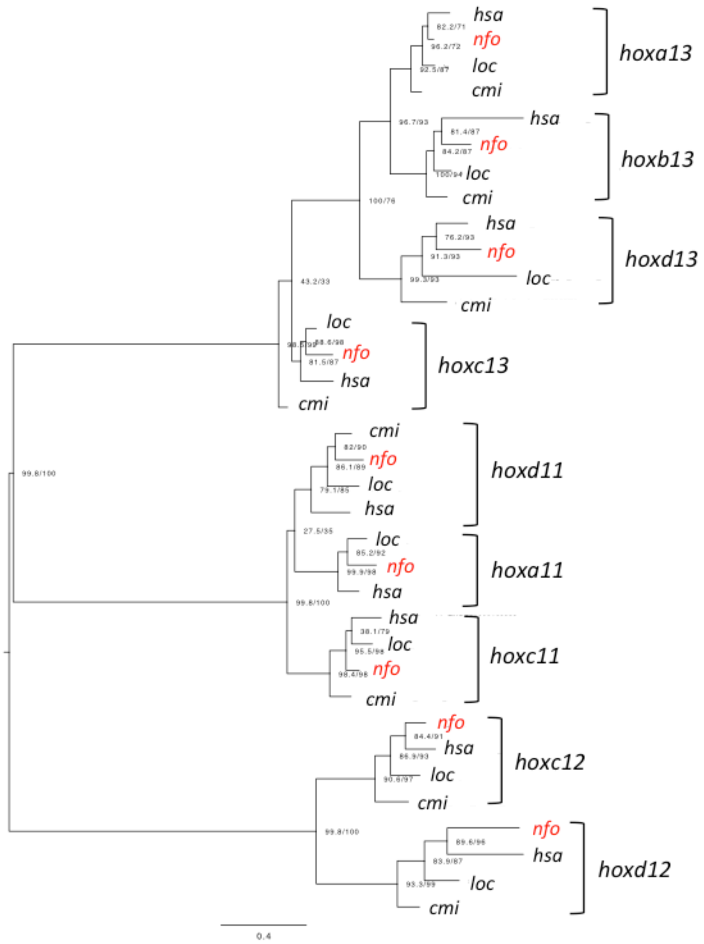

Fig. S5. Identification of posterior *hox* gene sequences in the Australian lungfish.
Gene tree confirming correct orthology assignment of the Australian lungfish (nfo) *hoxa13, hoxb13, hoxc13, hoxd13, hoxa11, hoxc11, hoxd11, hoxc12 and hoxd12* sequences (red) by comparison with the orthologous human (hsa), spotted gar (lco) and elephant shark (cmi) protein sequences. Branch lengths are expected replacements per site and numbers at nodes ultrafast bootstrap and SH-like aLRT support. Branch lengths are expected replacements per site and numbers at nodes ultrafast bootstrap and SH-like aLRT support.

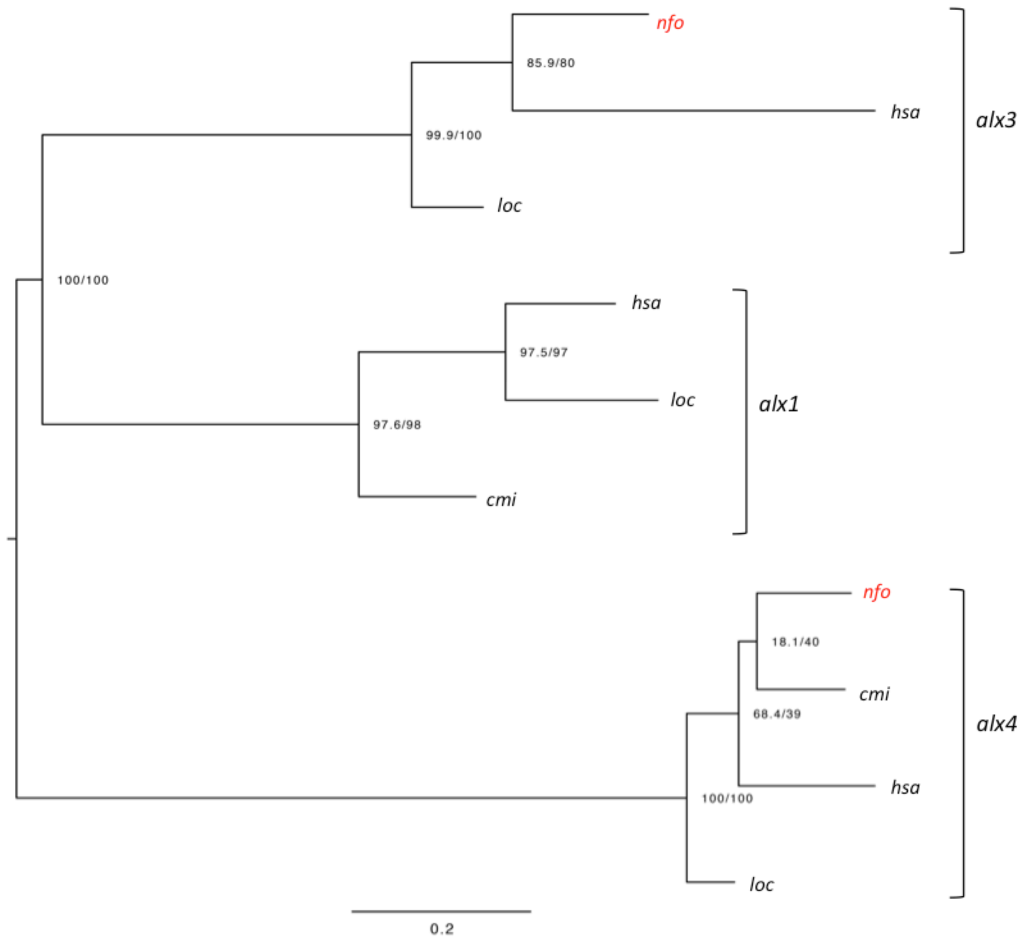

Fig. S6. Identification of *alx* gene sequences in the Australian lungfish.
Gene tree confirming correct orthology assignment of the Australian lungfish (nfo) *alx3* and *alx4* sequences (red) by comparison with the orthologous and paralogous (*alx1*) human (hsa), spotted gar (lco) and elephant shark (cmi) protein sequences. Branch lengths are expected replacements per site and numbers at nodes ultrafast bootstrap and SH-like aLRT support.

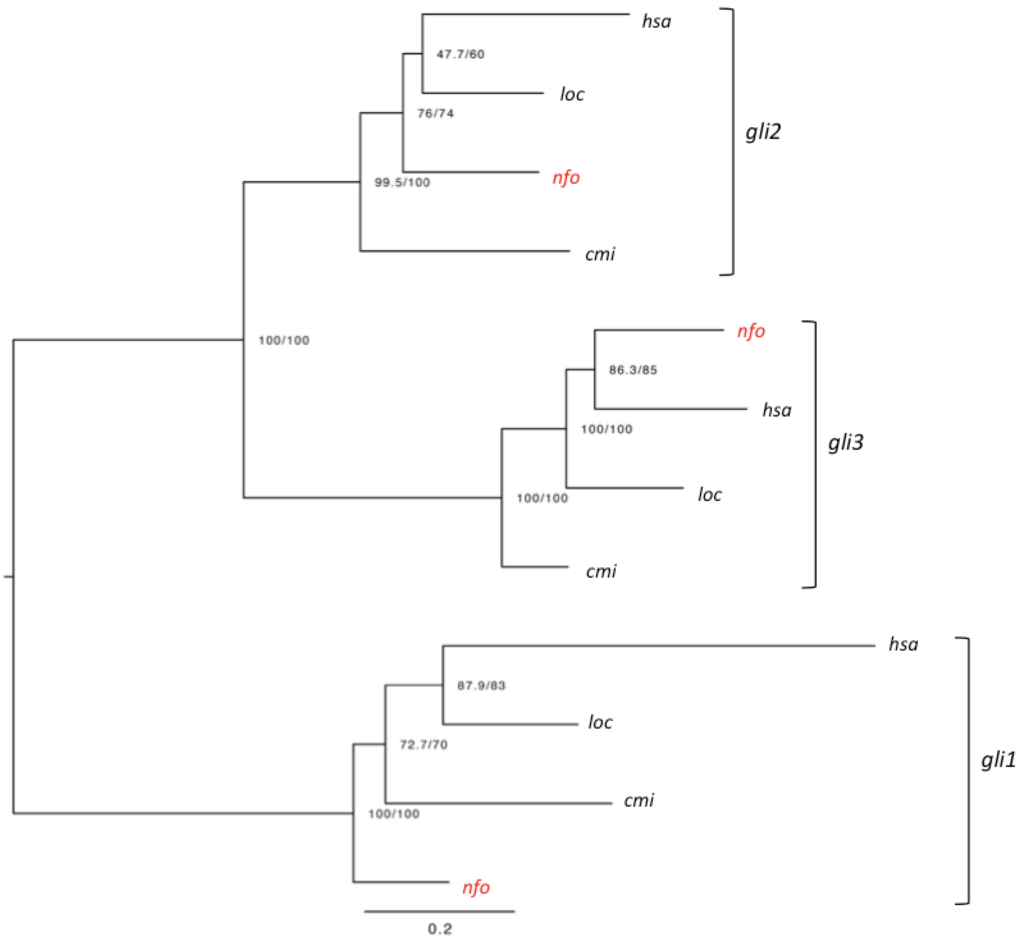

Fig. S7. **Identification of *gli* gene sequences in the Australian lungfish.**
Gene tree confirming correct orthology assignment of the Australian lungfish (nfo) *gli1, gli2* and *gli3* sequences (red) by comparison with the orthologous human (hsa), spotted gar (lco) and elephant shark (cmi) protein sequences. Branch lengths are expected replacements per site and numbers at nodes ultrafast bootstrap and SH-like aLRT support.

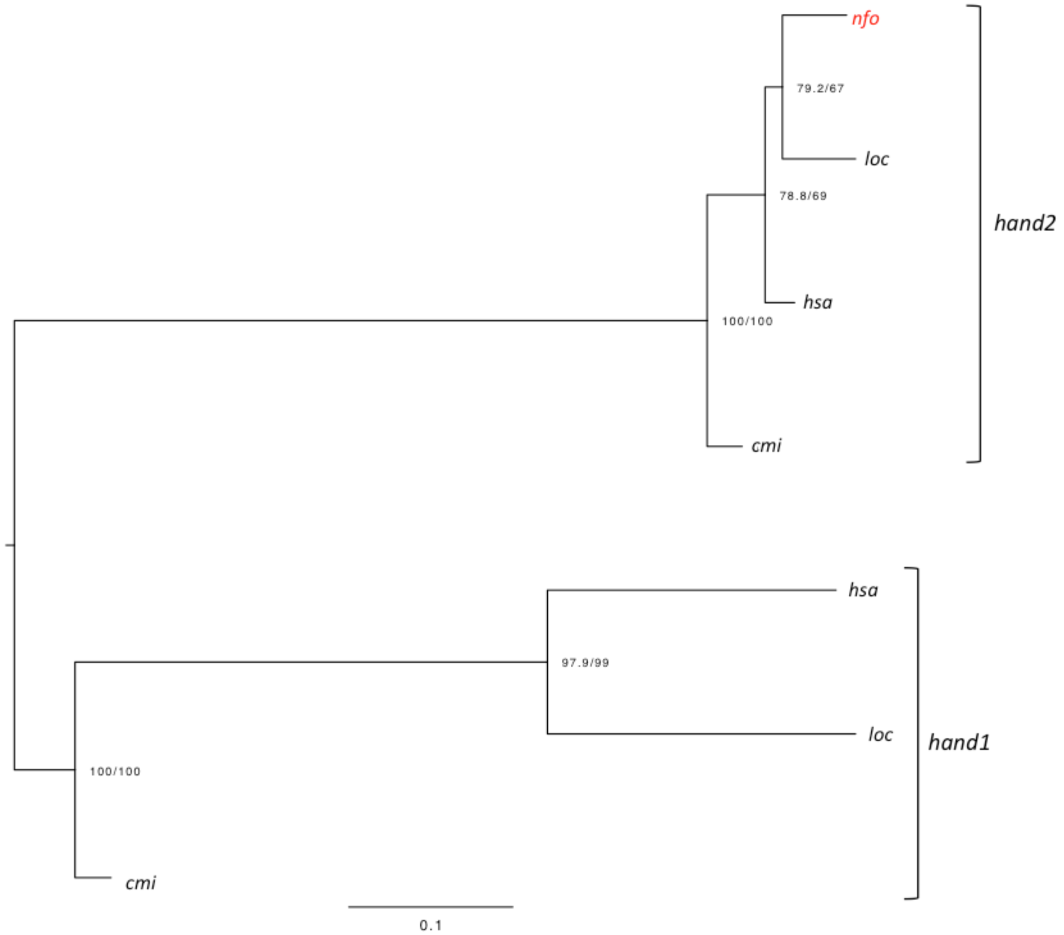

Fig. S8. **Identification of *hand2* in the Australian lungfish.**
Gene tree confirming correct orthology assignment of the Australian lungfish (nfo) *hand2* sequence (red) by comparison with the orthologous and paralogous (*hand1*) human (hsa), spotted gar (lco) and elephant shark (cmi) protein sequences. Branch lengths are expected replacements per site and numbers at nodes ultrafast bootstrap and SH-like aLRT support.

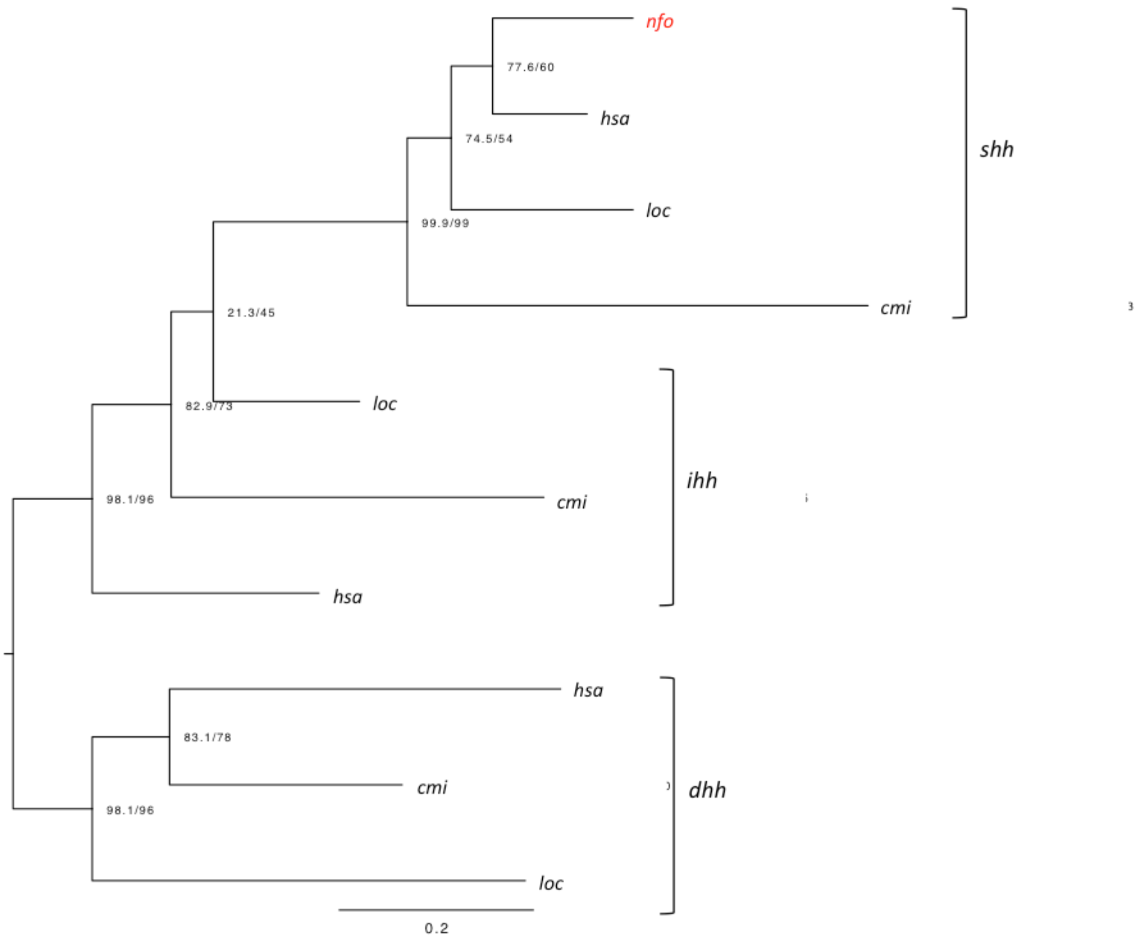

Fig. S9. Identification of *shh* in the Australian lungfish.
Gene tree confirming correct orthology assignment of the Australian lungfish (nfo) *shh* sequence (red) by comparison with the orthologous and paralogous (*ihh and dhh*) human (hsa), spotted gar (lco) and elephant shark (cmi) protein sequences. Branch lengths are expected replacements per site and numbers at nodes ultrafast bootstrap and SH-like aLRT support.

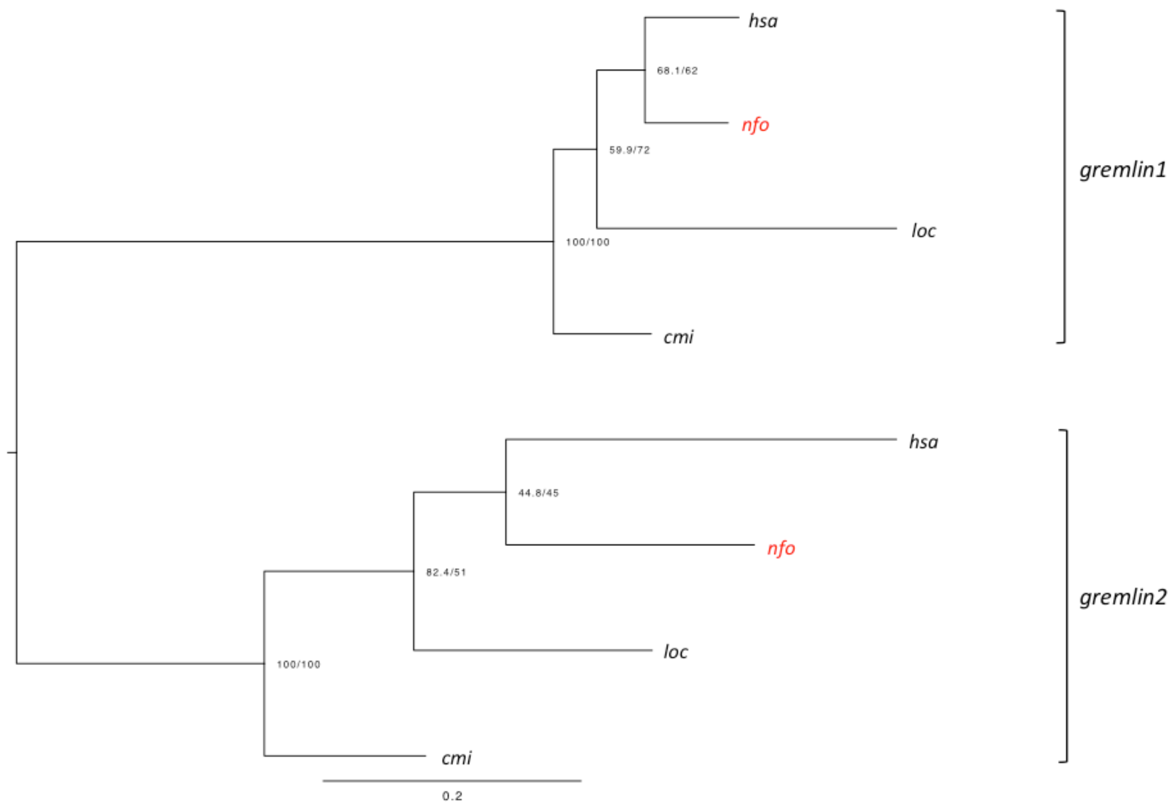

Fig. S10. **Identification of *gremlin* gene sequences in the Australian lungfish.**
Gene tree confirming correct orthology assignment of the Australian lungfish (nfo) *gremlin1* and *gremlin2* sequences (red) by comparison with the orthologous human (hsa), spotted gar (lco) and elephant shark (cmi) protein sequences. Branch lengths are expected replacements per site and numbers at nodes ultrafast bootstrap and SH-like aLRT support.

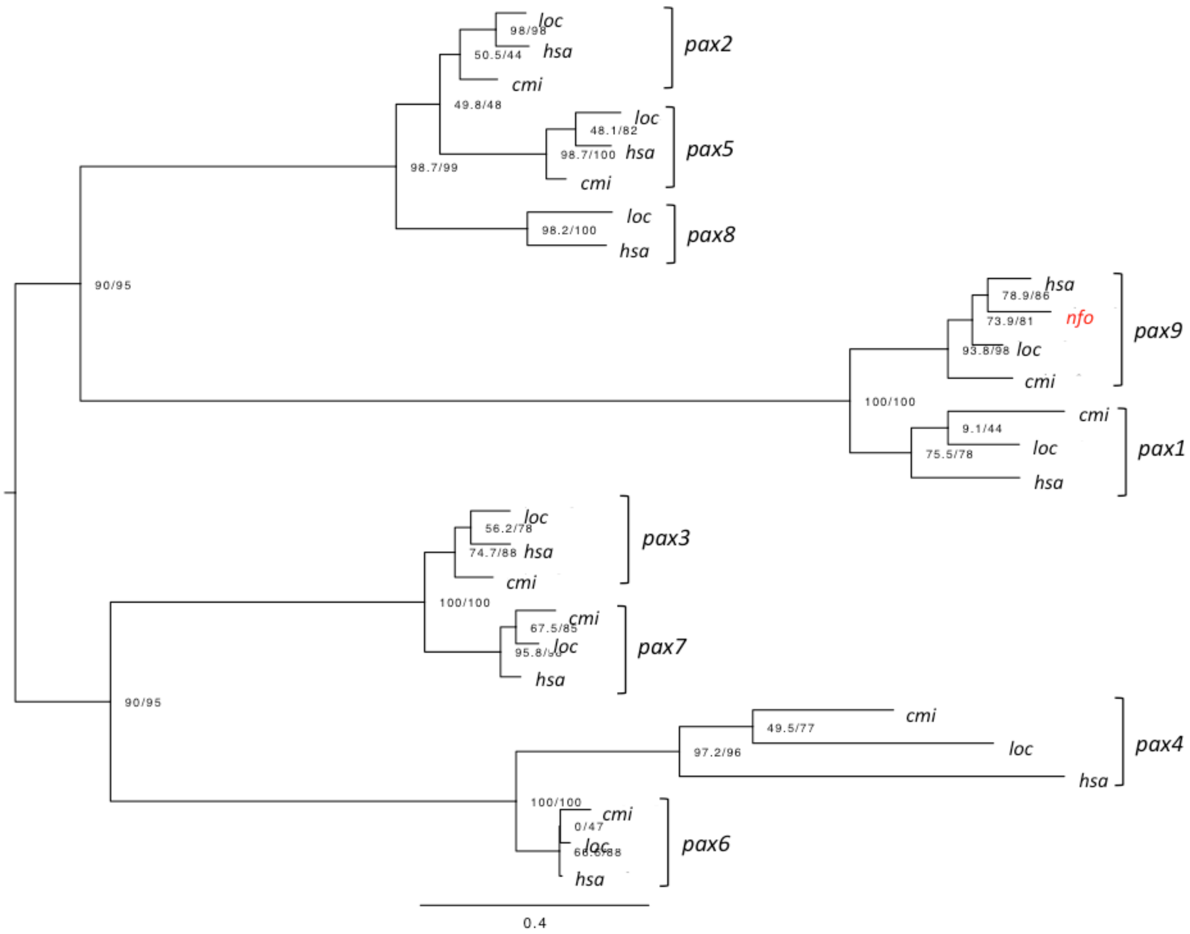

Fig. S11. Identification of *pax9* in the Australian lungfish.
Gene tree confirming correct orthology assignment of the Australian lungfish (nfo) *pax9* sequence (red) by comparison with the orthologous and paralogous (*pax1, pax2, pax3,pax4, pax5, pax6, pax7* and *pax8*) human (hsa), spotted gar (lco) and elephant shark (cmi) protein sequences. Branch lengths are expected replacements per site and numbers at nodes ultrafast bootstrap and SH-like aLRT support.

Table S1. Primer sequences used for cloning of probes. If multiple probe sequences were cloned their relative position within the gene is indicated with 5' or 3' respectively. The probes used in Fig.S1 are indicated with reference to this figure.

Neoceratodus forsteri	**Forward primer**	**Reverse primer**
hoxa13 5'	ATGACAGCGTCAGTGCTCCTTC	GCAGCGTGGATTTCCAGAGGTG
hoxa13 3'	CAGATGTCGTGTCGCATCCTTC	AATGAAAGACAAATTAGCTTGC
hoxd13 5'	AACTCGTCTCACGGAATGTTAC	TAAATGCGACATCCCCTGCAT
hoxd13 3'	GGGATGTCGCATTTAGTCAAC	CTAAGGGTCTCAACCAATTCAG
hand2 5'	GTTTTCCTCACCACCCAGT	GGAATGCTTTTCTTCAAATATCC
alx4 5'	GAGAAGGTCTTCCAGAAAACACAC	CCATATCATGGGCTACATGAC
alx4 3'	CCATCAACAGTTATGACCTGAATG	CAGAACTCCACGGTGGGTGAAC
hoxd11 5'	ATGACCGAAATCGATGATCG	TTGTGCTACTGCTCTTCTCGGAAG
hoxd11 3'	GGGATGTCGCATTTAGTCAAC	CTAAGGGTCTCAACCAATTCAG
col2a1 5'	CAAGATGGTCTTCCAGGTCCCAAG	CTGTTCTCCTTTAATACCTGGTTG
col2a1 3'	ACCAGGTCCTCAAGGTCCTTC	GGTTGACCAGGAGGACCAGATG
actinodin1/2 5'	CACACCTTACCCCATGACTG	TGTAGGGGTCTGAGCCATAC
actinodin1/2 3'	CACACCTTACCCCATGACTG	TAATTACCAGTGGGAGAGCAG
gli3 5'	GTTCTCAACCAAGCATCTGC	TATCATGCTGCTGGATTGTCTG
gli3 3'	GATAATAATCCATACCATGTGCAC	TGGAATGTGAGAGACACTCGTG
shh	GGGGCCAGTGGCAGATATGAAG	ATAGCTCATCCTTAGAGGTGCAA
gremlin1	ATGATCCGCCTGATGTATGCTT	CATATAAAGAAATGGGTGACTATG
pax9	CACCAAATGGACTACCAGCAG	ATGAGATGTTCAAGCTTCCAC
hoxa13 (Fig.S1)	CAAGGCTTGGATGAAATGAA	TGCAGGACTTGATAGCGTTG
hoxd13 (Fig.S1)	GCCAAAGAACTTGCTTTTTACC	CTTGTCTTTCAGACAGGTTGGTAG
hand2 (Fig.S1)	CACCCNGTRRTKCACCATG	TCCATRAGGTARGCKATRTARCTGG
gremlin1 (Fig.S1)	GWTYHCARGGMGCYATYCCTC	TGTAGAARGARTTGCAYTGBCC
Astatotilapia burtoni		
alx4a	ACGCTCATCTCCTGCTATGGAAA	GAGATCATAGCCATTGATTCCCCCA
hand2	GTTCTTCCCGAGCCTCCGTC	GCGTATTTTTGTGTGTCACTATG

REFERENCES AND NOTES

1. J. M. Woltering, D. Duboule, The origin of digits: Expression patterns versus regulatory mechanisms. *Dev. Cell* **18**, 526–532 (2010).
2. G. P. Wagner, C. H. Chiu, The tetrapod limb: A hypothesis on its origin. *J. Exp. Zool.* **291**, 226–240 (2001).
3. T. S. Westoll, The origin of the primitive tetrapod limb. *Proc. R. Soc. Lond. Series B Biol. Sci.* **131**, 373–393 (1943).
4. W. Gregory, H. Raven, PartIII: On the transformation of the pectoral and pelvic paddles of *Eusthenopteron* type into pentadatylate limbs. *Ann. New York Acad. Sci.* **42**, 313–327 (1941).
5. F. Langellotto, M. Fiorentino, E. De Felice, L. Caputi, V. Nittoli, J. M. P. Joss, P. Sordino, Expression of *meis* and *hoxa11* in dipnoan and teleost fins provides new insights into the evolution of vertebrate appendages. *Evodevo* **9**, 11 (2018).
6. C. A. Boisvert, E. Mark-Kurik, P. E. Ahlberg, The pectoral fin of *Panderichthys* and the origin of digits. *Nature* **456**, 636–638 (2008).
7. N. H. Shubin, E. B. Daeschler, F. A. Jenkins Jr., The pectoral fin of *Tiktaalik roseae* and the origin of the tetrapod limb. *Nature* **440**, 764–771 (2006).
8. Z. Johanson, J. Joss, C. A. Boisvert, R. Ericsson, M. Sutija, P. E. Ahlberg, Fish fingers: Digit homologues in sarcopterygian fish fins. *J. Exp. Zool. B Mol. Dev. Evol.* **308**, 757–768 (2007).
9. M. I. Coates, J. E. Jeffery, M. Rut, Fins to limbs: What the fossils say. *Evol. Dev.* **4**, 390–401 (2002).
10. M. Tanaka, Fins into limbs: Autopod acquisition and anterior elements reduction by modifying gene networks involving *5'Hox, Gli3,* and *Shh*. *Dev. Biol.* **413**, 1–7 (2016).
11. G. Wagner, H. Larsson, Fins and limbs in the study of evolutionary novelties, in *Fins into Limbs: Evolution, Development, and Transformation*, B. Hall, Ed. (The Univ. of Chicago Press, Chicago, 2007), chap. 4, pp. 49–61.
12. D. Ahn, R. K. Ho, Tri-phasic expression of posterior *Hox* genes during development of pectoral fins in zebrafish: Implications for the evolution of vertebrate paired appendages. *Dev. Biol.* **322**, 220–233 (2008).
13. P. Sordino, F. van der Hoeven, D. Duboule, *Hox* gene expression in teleost fins and the origin of vertebrate digits. *Nature* **375**, 678–681 (1995).

14. J. M. Woltering, D. Noordermeer, M. Leleu, D. Duboule, Conservation and divergence of regulatory strategies at *Hox* Loci and the origin of tetrapod digits. *PLOS Biol.* **12**, e1001773 (2014).
15. R. Freitas, C. Gomez-Marin, J. M. Wilson, F. Casares, J. L. Gomez-Skarmeta, *Hoxd13* contribution to the evolution of vertebrate appendages. *Dev. Cell* **23**, 1219–1229 (2012).
16. W. Masselink, N. J. Cole, F. Fenyes, S. Berger, C. Sonntag, A. Wood, P. D. Nguyen, N. Cohen, F. Knopf, G. Weidinger, T. E. Hall, P. D. Currie, A somitic contribution to the apical ectodermal ridge is essential for fin formation. *Nature* **535**, 542–546 (2016).
17. J. Zhang, P. Wagh, D. Guay, L. Sanchez-Pulido, B. K. Padhi, V. Korzh, M. A. Andrade-Navarro, M.-A. Akimenko, Loss of fish actinotrichia proteins and the fin-to-limb transition. *Nature* **466**, 234–237 (2010).
18. M. C. Davis, R. D. Dahn, N. H. Shubin, An autopodial-like pattern of Hox expression in the fins of a basal actinopterygian fish. *Nature* **447**, 473–476 (2007).
19. T. Nakamura, A. R. Gehrke, J. Lemberg, J. Szymaszek, N. H. Shubin, Digits and fin rays share common developmental histories. *Nature* **537**, 225–228 (2016).
20. I. Irisarri, A. Meyer, The identification of the closest living relative(s) of tetrapods: Phylogenomic lessons for resolving short ancient internodes. *Syst. Biol.* **65**, 1057–1075 (2016).
21. C. E. Nelson, B. A. Morgan, A. C. Burke, E. Laufer, E. DiMambro, L. C. Murtaugh, E. Gonzales, L. Tessarollo, L. F. Parada, C. Tabin, Analysis of Hox gene expression in the chick limb bud. *Development* **122**, 1449–1466 (1996).
22. F. J. Tulenko, J. L. Massey, E. Holmquist, G. Kigundu, S. Thomas, S. M. E. Smith, S. Mazan, M. C. Davis, Fin-fold development in paddlefish and catshark and implications for the evolution of the autopod. *Proc. Biol. Sci.* **284**, 20162780 (2017).
23. J. M. Woltering, M. Holzem, A. Meyer, Lissamphibian limbs and the origins of tetrapod *hox* domains. *Dev. Biol.* **456**, 138–144 (2019).
24. R. Zeller, J. López-Ríos, A. Zuniga, Vertebrate limb bud development: Moving towards integrative analysis of organogenesis. *Nat. Rev. Genet.* **10**, 845–858 (2009).
25. M. Fernandez-Teran, M. E. Piedra, I. S. Kathiriya, D. Srivastava, J. C. Rodriguez-Rey, M. A. Ros, Role of dHAND in the anterior-posterior polarization of the limb bud: Implications for the Sonic hedgehog pathway. *Development* **127**, 2133–2142 (2000).

26. B. Tarchini, D. Duboule, Control of *Hoxd* genes' collinearity during early limb development. *Dev. Cell* **10**, 93–103 (2006).
27. B. A. Firulli, D. Krawchuk, V. E. Centonze, N. Vargesson, D. M. Virshup, S. J. Conway, P. Cserjesi, E. Laufer, A. B. Firulli, Altered Twist1 and Hand2 dimerization is associated with Saethre-Chotzen syndrome and limb abnormalities. *Nat. Genet.* **37**, 373–381 (2005).
28. K. Kawahata, I. R. Cordeiro, S. Ueda, G. Sheng, Y. Moriyama, C. Nishimori, R. Yu, M. Koizumi, M. Okabe, M. Tanaka, Evolution of the avian digital pattern. *Sci. Rep.* **9**, 8560 (2019).
29. M. Takahashi, K. Tamura, D. Büscher, H. Masuya, S. Yonei-Tamura, K. Matsumoto, M. Naitoh-Matsuo, J. Takeuchi, K. Ogura, T. Shiroishi, T. Ogura, J. C. Izpisua Belmonte, The role of Alx-4 in the establishment of anteroposterior polarity during vertebrate limb development. *Development* **125**, 4417–4425 (1998).
30. E. McGlinn, K. L. van Bueren, S. Fiorenza, R. Mo, A. M. Poh, A. Forrest, M. B. Soares, M. de Fatima Bonaldo, S. Grimmond, C.-C. Hui, B. Wainwright, C. Wicking, *Pax9* and *Jagged1* act downstream of Gli3 in vertebrate limb development. *Mech. Dev.* **122**, 1218–1233 (2005).
31. S. Kuijper, H. Feitsma, R. Sheth, J. Korving, M. Reijnen, F. Meijlink, Function and regulation of *Alx4* in limb development: Complex genetic interactions with *Gli3* and *Shh*. *Dev. Biol.* **285**, 533–544 (2005).
32. P. te Welscher, M. Fernandez-Teran, M. A. Ros, R. Zeller, Mutual genetic antagonism involving GLI3 and dHAND prepatterns the vertebrate limb bud mesenchyme prior to SHH signaling. *Genes Dev.* **16**, 421–426 (2002).
33. P. te Welscher, A. Zuniga, S. Kuijper, T. Drenth, H. J. Goedemans, F. Meijlink, R. Zeller, Progression of vertebrate limb development through SHH-mediated counteraction of GLI3. *Science* **298**, 827–830 (2002).
34. L. Panman, A. Galli, N. Lagarde, O. Michos, G. Soete, A. Zuniga, R. Zeller, Differential regulation of gene expression in the digit forming area of the mouse limb bud by SHH and gremlin 1/FGF-mediated epithelial-mesenchymal signalling. *Development* **133**, 3419–3428 (2006).
35. Y. Litingtung, R. D. Dahn, Y. Li, J. F. Fallon, C. Chiang, *Shh* and *Gli3* are dispensable for limb skeleton formation but regulate digit number and identity. *Nature* **418**, 979–983 (2002).

36. M. F. Bastida, R. Pérez-Gómez, A. Trofka, J. Zhu, A. Rada-Iglesias, R. Sheth, H. S. Stadler, S. Mackem, M. A. Ros, The formation of the thumb requires direct modulation of *Gli3* transcription by Hoxa13. *Proc. Natl. Acad. Sci. U.S.A.* **117**, 1090–1096 (2020).
37. P. J. Scherz, B. D. Harfe, A. P. McMahon, C. J. Tabin, The limb bud Shh-Fgf feedback loop is terminated by expansion of former ZPA cells. *Science* **305**, 396–399 (2004).
38. L. Niswander, S. Jeffrey, G. R. Martin, C. Tickle, A positive feedback loop coordinates growth and patterning in the vertebrate limb. *Nature* **371**, 609–612 (1994).
39. V. S. Hodgkinson, R. Ericsson, Z. Johanson, J. M. P. Joss, The apical ectodermal ridge in the pectoral fin of the Australian Lungfish (*Neoceratodus forsteri*): Keeping the fin to limb transition in the fold. *Acta Zoologica* **90**, 253–263 (2009).
40. K. Sakamoto, K. Onimaru, K. Munakata, N. Suda, M. Tamura, H. Ochi, M. Tanaka, Heterochronic shift in *Hox*-mediated activation of *sonic hedgehog* leads to morphological changes during fin development. *PLOS ONE* **4**, e5121 (2009).
41. K. Onimaru, S. Kuraku, W. Takagi, S. Hyodo, J. Sharpe, M. Tanaka, A shift in anterior-posterior positional information underlies the fin-to-limb evolution. *eLife* **4**, e07048 (2015).
42. F. J. Tulenko, G. J. Augustus, J. L. Massey, S. E. Sims, S. Mazan, M. C. Davis, HoxD expression in the fin-fold compartment of basal gnathostomes and implications for paired appendage evolution. *Sci. Rep.* **6**, 22720 (2016).
43. G. Nachtrab, K. Kikuchi, V. A. Tornini, K. D. Poss, Transcriptional components of anteroposterior positional information during zebrafish fin regeneration. *Development* **140**, 3754–3764 (2013).
44. T. Montavon, N. Soshnikova, B. Mascrez, E. Joye, L. Thevenet, E. Splinter, W. de Laat, F. Spitz, D. Duboule, A regulatory archipelago controls *Hox* genes transcription in digits. *Cell* **147**, 1132–1145 (2011).
45. N. Holmgren, On the origin of the tetrapod limb. *Acta Zoologica* **14**, 185–295 (1933).
46. L. Beccari, N. Yakushiji-Kaminatsui, J. M. Woltering, A. Necsulea, N. Lonfat, E. Rodríguez-Carballo, B. Mascrez, S. Yamamoto, A. Kuroiwa, D. Duboule, A role for HOX13 proteins in the regulatory switch between TADs at the *HoxD* locus. *Genes Dev.* **30**, 1172–1186 (2016).
47. R. Sheth, I. Barozzi, D. Langlais, M. Osterwalder, S. Nemec, H. L. Carlson, H. S. Stadler, A. Visel, J. Drouin, M. Kmita, Distal limb patterning requires modulation of *cis*-regulatory activities by HOX13. *Cell Rep.* **17**, 2913–2926 (2016).

48. N. Shubin, P. Alberg, A Morphogenetic Approach to the Origin and Basic Organization of the Tetrapod Limb, in *Evolutionary Biology*, M. K. Hecht, B. Wallace, G. T. Prance, Eds. (Springer, Boston, MA, 1986).
49. M. J. Cohn, C. O. Lovejoy, L. Wolpert, M. I. Coates, Branching, segmentation and the metapterygial axis: Pattern versus process in the vertebrate limb. *Bioessays* **24**, 460–465 (2002).
50. R. Cloutier, A. M. Clement, M. S. Y. Lee, R. Noël, I. Béchard, V. Roy, J. A. Long, *Elpistostege* and the origin of the vertebrate hand. *Nature* **579**, 549–554 (2020).
51. J. M. Woltering, F. J. Vonk, H. Müller, N. Bardine, I. L. Tuduce, M. A. G. de Bakker, W. Knöchel, I. O. Sirbu, A. J. Durston, M. K. Richardson, Axial patterning in snakes and caecilians: Evidence for an alternative interpretation of the *Hox* code. *Dev. Biol.* **332**, 82–89 (2009).
52. A. Kemp, The embryological development of the queensland lungfish, *Neoceratodus forsteri* (Krefft). *Mem. Queensl. Mus.* **20**, 553–597 (1982).
53. J. M. Woltering, M. Holzem, R. F. Schneider, V. Nanos, A. Meyer, The skeletal ontogeny of *Astatotilapia burtoni* – A direct-developing model system for the evolution and development of the teleost body plan. *BMC Dev. Biol.* **18**, 8 (2018).
54. G. Lauter, I. Söll, G. Hauptmann, Multicolor fluorescent *in situ* hybridization to define abutting and overlapping gene expression in the embryonic zebrafish brain. *Neural Dev.* **6**, 10 (2011).
55. H. L. Sive, R. M. Grainger, R. M. Harland, *Early development of Xenopus laevis: A Laboratory Manual* (Cold Spring Harbor Laboratory Press, 2000).
56. D. Bader, T. Masaki, D. A. Fischman, Immunochemical analysis of myosin heavy chain during avian myogenesis in vivo and in vitro. *J. Cell Biol.* **95**, 763–770 (1982).
57. A. M. Bolger, M. Lohse, B. Usadel, Trimmomatic: A flexible trimmer for Illumina sequence data. *Bioinformatics* **30**, 2114–2120 (2014).
58. R. Schmieder, R. Edwards, Quality control and preprocessing of metagenomic datasets. *Bioinformatics* **27**, 863–864 (2011).
59. M. G. Grabherr, B. J. Haas, M. Yassour, J. Z. Levin, D. A. Thompson, I. Amit, X. Adiconis, L. Fan, R. Raychowdhury, Q. Zeng, Z. Chen, E. Mauceli, N. Hacohen, A. Gnirke, N. Rhind, F. di Palma, B. W. Birren, C. Nusbaum, K. Lindblad-Toh, N. Friedman, A. Regev, Full-

length transcriptome assembly from RNA-Seq data without a reference genome. *Nat. Biotechnol.* **29**, 644–652 (2011).

60. S. Whelan, I. Irisarri, F. Burki, PREQUAL: Detecting non-homologous characters in sets of unaligned homologous sequences. *Bioinformatics* **34**, 3929–3930 (2018).
61. K. Katoh, D. M. Standley, MAFFT multiple sequence alignment software version 7: Improvements in performance and usability. *Mol. Biol. Evol.* **30**, 772–780 (2013).
62. L.-T. Nguyen, H. A. Schmidt, A. von Haeseler, B. Q. Minh, IQ-TREE: A fast and effective stochastic algorithm for estimating maximum-likelihood phylogenies. *Mol. Biol. Evol.* **32**, 268–274 (2015).
